# Supplementary material for: Transdermal Delivery of Lidocaine-Loaded Elastic Nano-Liposomes with Microneedle Array Pretreatment
Source: Biomedicines. 2021 May 23;9(6):592. doi: 10.3390/biomedicines9060592 (PMC8224805; doi:10.3390/biomedicines9060592)
Supplement: Supplementary file 1 [file biomedicines-09-00592-s001.zip › biomedicines-1216815-supplementary.pdf]

Supplementary Material

Transdermal Delivery of Lidocaine-Loaded Elastic Nano-liposomes with Microneedle Array Pretreatment

Yang Liu [1], Maosen Cheng [1], Junqi Zhao [1], Xiaoying Zhang [1], Zhen Huang [1], Yuhui Zang [1], Ying Ding [2,*], Junfeng Zhang [1] and Zhi Ding[1,3,*]

[1] State Key Laboratory of Pharmaceutical Biotechnology, School of Life Sciences, Nanjing University, Nanjing 210023, China

[2] Department of Anesthesiology, The Second Affiliated Hospital of Nanjing Medical University, Nanjing 210011, China

[3] Changzhou High-Tech Research Institute of Nanjing University, Changzhou, 213164, China

[*]**Correspondence to:**

Ying Ding, Department of Anesthesiology, the Second Affiliated Hospital of Nanjing Medical University, Jiangjiayuan 121, Nanjing 210011, China

Email: dingying.anesthesiology@gmail.com

Tel: +86 25 58509855

Zhi Ding, State Key Laboratory of Pharmaceutical Biotechnology, School of Life Sciences, Nanjing University, Xianlin Avenue 163, Nanjing 210023, China

E-mail: dingzhi@nju.edu.cn

Tel: +86 25 89681321

Fax: +86 25 89684060

S1. SPC/cholesterol mass ratio and pH effect in EE

To optimize the composition of ENL, SPC and cholesterol were selected to prepare

liposomes at 10:1, 10:2, and 10:3 (w/w) mass ratios and dissolved in trichloromethane as the organic phase. The following steps of LidH ENL preparation are the same as those described in the Materials and Methods section. The particle size of ENL and the EE of LidH were measured.

Next, 1× PBS at a pH of 5.5, 8.0, and 10.0 was used as the aqueous solution to dissolve the LidH powder. SPC and cholesterol (10:2, w/w) were dissolved in trichloromethane and mixed with 1× PBS LidH solutions at different pH values. The liposomes were prepared by reverse evaporation as described in the Methods and Materials section. The collected samples were then extruded through a 220 nm filter. The particle sizes of the ENLs and EE of LidH were measured.

Regarding the effect of the phospholipid composition, LidH at the concentration of 2% (w/w) and SPC/cholesterol at a mass ratio of 10:2 showed the best result of 33.49% ± 2.040% for trapping efficiency (Figure S1a). The increase in the incorporation of cholesterol for liposome preparation did not lead to a higher EE of LidH.

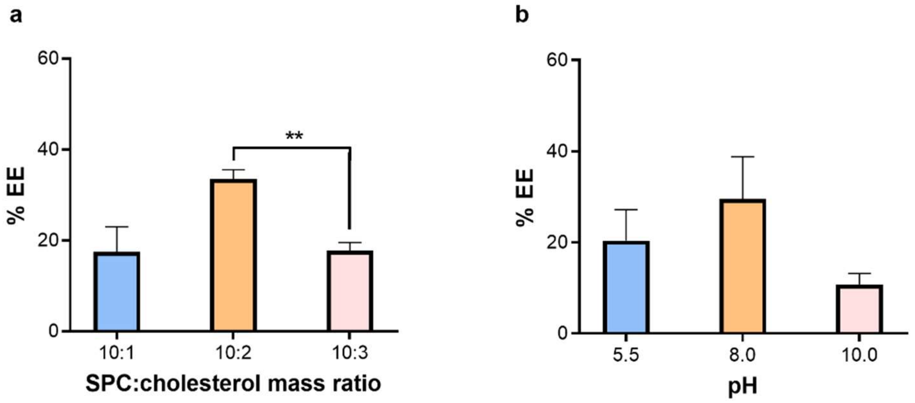

Figure S1 EE of LidH in different formulations. (**a**) Different SPC/cholesterol mass ratios: 10:1, 10:2, and 10:3. (**b**) Different pH values of aqueous phase: 5.5, 8.0, and 10.0. Data are shown as mean ± SD, n = 3. (**: $p < 0.01$, Student's *t*-test). Abbreviations: EE, encapsulation efficiency; LidH, lidocaine hydrochloride; SPC, soybean phosphatidylcholine.

Differences among groups with diverse pH values were not statistically significant (Figure S1b). pH 8.0 was selected since it exhibited a higher EE of 29.614% than that at

pH 5.5 and 10.0.

S2. Optimization of the sonication process

Extended sonication was applied to reduce liposome particle size (Figure S2). With 3 min sonication, the cumulative amount of permeated LidH was significantly higher than that without sonication ($p < 0.01$). Although there was no significant difference in the cumulative amount of permeated LidH between 1 and 3 min sonication after 6 h, in the first hour, cumulative LidH permeation was 1.6 times higher (792.4 μg/cm^2) with 3 min sonication than with 1 min sonication ($p < 0.01$). Thus, 3 min sonication was chosen to reduce the particle size at the end step of ENL preparation. In our study, the particle size of Span 80 ENL reduced from 205.2 ± 30.60 nm to 155.43 ± 7.42 nm after sonication.

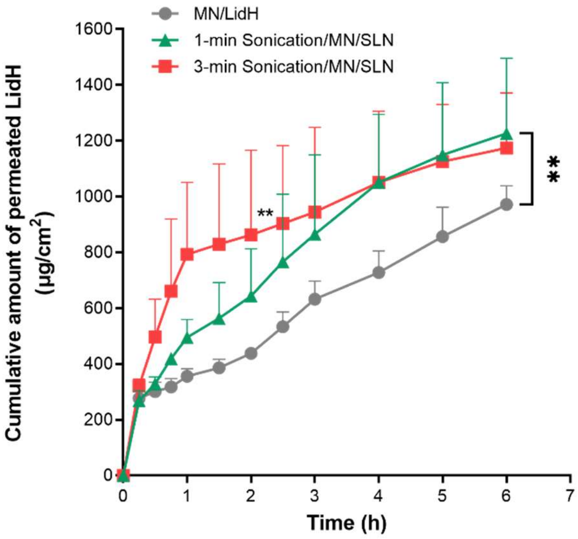

Figure S2 Cumulative permeation of LidH with 1 or 3 min sonication at the end step of SLN preparation. Data are shown as mean ± SD, n = 3. ** indicates statistical significance at $p < 0.01$ using the Student's *t*-test. Abbreviations: LidH, lidocaine hydrochloride; MN, microneedle; SLN, solid lipid nanoparticle.
